# Using volunteered observations to map human exposure to ticks

**DOI:** 10.1038/s41598-018-33900-2

**Published:** 2018-10-18

**Authors:** Irene Garcia-Marti, Raul Zurita-Milla, Margriet G. Harms, Arno Swart

**Affiliations:** 1 Department of Geo-Information Processing, Faculty of Geo-Information and Earth Observation (ITC), University of Twente, Enschede, The Netherlands; 2 Centre for Infectious Disease Control, National Institute for Public Health and the Environment (RIVM), Bilthoven, The Netherlands

## Abstract

Lyme borreliosis (LB) is the most prevalent tick-borne disease in Europe and its incidence has steadily increased over the last two decades. In the Netherlands alone, more than 20,000 citizens are affected by LB each year. Because of this, two Dutch citizen science projects were started to monitor tick bites. Both projects have collected nearly 50,000 geo-located tick bite reports over the period 2006–2016. The number of tick bite reports per area unit is a proxy of tick bite risk. This risk can also be modelled as the result of the interaction of hazard (e.g. tick activity) and human exposure (e.g. outdoor recreational activities). Multiple studies have focused on quantifying tick hazard. However, quantifying human exposure is a harder task. In this work, we make a first step to map human exposure to ticks by combining tick bite reports with a tick hazard model. Our results show human exposure to tick bites in all forested areas of the Netherlands. This information could facilitate the cooperation between public health specialists and forest managers to create better mitigation campaigns for tick-borne diseases, and it could also support the design of improved plans for ecosystem management.

## Introduction

Forests are complex dynamic systems that provide a wide array of ecosystem services to society, such as groundwater protection, and wood and fiber production^1^. Also, forests provide recreational services (e.g. sports, leisure activities), which may have positive (e.g. stress reduction) and negative (e.g. increased exposure to pathogens) impacts on human health^2^. The transmission of pathogens causing tick-borne diseases to human hosts poses an important threat to public health^3^. In this regard, the European Centre for Disease Prevention and Control surveys five different tick-borne diseases (i.e. Lyme borreliosis, tick-borne encephalitis, relapsing fever, Crimean-Congo hemorrhagic fever, and Mediterranean spotted fever, from https://ecdc.europa.eu/en/publications-data/presentation-tick-borne-diseases-healthcare-professionals, last accessed July 5^th^, 2018), and reports the prevalence of these diseases across the European continent.

The incidence of Lyme borreliosis (LB), the most prevalent tick-borne disease in Europe, has steadily increased during the period 1990–2010 in, at least, nine European countries^4^. In recent years, however, sub European sentinel networks of general practitioners have identified the first signs of stabilization^5–7^. In the Netherlands alone, the number of LB cases has continuously increased since the mid-1990s^8,9^, but a recent study shows that this trend is also stabilizing^10^. Yet, over 20,000 citizens are diagnosed each year with LB, a situation that prompted researchers from Wageningen University and the Dutch Institute for Public Health and the Environment (RIVM), to start two crowdsourced projects to collect data on tick bites. Since 2006, the platforms Natuurkalender (NK; “nature’s calendar”; http://www.natuurkalender.nl) and Tekenradar (TR; “tick radar”; https://www.tekenradar.nl/), have collected nearly 50,000 geo-located tick bite reports. To the best of our knowledge, these platforms constitute the first citizen science projects that specifically focus on ticks and tick-borne diseases. Citizen science projects present an interesting characteristic when compared to classical forms of data acquisition (e.g. ground surveys or sensor networks): the ubiquity of the crowd allows a fine-grained sampling in space and time, which makes it possible to monitor elusive public health threats, such as tick bites.

The volunteered tick bite reports collected by the NK and TR projects depict the risk of getting a tick bite. This risk is the result of the interaction of two components: hazard (e.g. tick activity) and human exposure (e.g. recreational intensity in a location)^11^, which tends to occur outdoors (e.g. forests, urban parks). There are significant efforts in literature to quantify the hazard component^12–14^, but finding proxies of exposure is a harder task, due to the unavailability of human recreational metrics at the national scale. Quantifying recreational pressure in nature is of interest in fields as diverse as public health, forestry management or environmental science. In public health, knowing the intensity of recreational activities might help delimiting locations that serve as an interface between natural elements and humans^13,15–17^. This can be useful to better design tick bite prevention campaigns in forested areas. However, the complexity of forest ownership in the Netherlands might pose hurdles to this task. The sixth Dutch National Forest Inventory^18^ indicates that forests occupy 373,480 hectares (roughly, 11% of the territory). Public organizations (e.g. Staatbosbeheer: https://www.staatsbosbeheer.nl/, municipalities) own 260,900 hectares of forest, whereas private partners (e.g. Natuurmonumenten: https://www.natuurmonumenten.nl/, citizens) own 112,600 hectares of forest. Dutch law requires private owners with a property larger than 5 hectares to be registered at the Dutch Industrial Board for Forest and Nature (i.e. Bosschap http://www.bosschap.nl/). Currently, 1,431 citizens own 61,000 hectares of forest larger than 5 hectares, and an unknown number of citizens own 51,600 hectares of smaller forest patches^18,19^. Thus, educating a relatively small group of public and private foresters to reduce tick bites in their domain, might lead to a substantial decrease in the number of LB cases per year. For instance, forest owners with parcels presenting a medium or high human exposure, could implement preventive measures of tick habitat manipulation, such as grass mowing, removing leaf litter, or covering heavily visited locations with dry substrates (e.g. wood chips, gravel)^20^.

In this work, we present a novel method to quantify country-wide exposure to ticks in forested areas. Our method is based on volunteered tick bite reports, and on a hazard model developed in our previous work^12^. This hazard model was also based on volunteered data about tick activity in forested areas. The resulting human exposure is presented as a categorical map that shows the recreational intensity across Dutch forested areas. Such a human exposure map should facilitate the cooperation between public health specialists and forest managers to jointly tackle public awareness about tick bite prevention, especially in locations with a high intensity of human exposure. In addition, this first-of-its-kind exposure map helps to understand and locate sources of potential landscape disturbance (by visitors) and could support better ecological management practices.

## Modelling Human Exposure to Tick Bites

Obtaining measures of human exposure to ticks is a challenging task due to the unavailability of nation-wide datasets representing human activities outdoors. However, human exposure is tightly related to risk and hazard, so it can be calculated from these two variables. In this section we describe the theoretical background, operationalisation and the data processing workflow that allows the calculation of human exposure and graphical mapping of this variable.

### Theoretical background

In the field of risk assessment, risk (R) is often modelled as a function of hazard (H) and exposure (E). The relationship between the three variables can be conceptualized as R = H × E^11^. In the paragraphs below, we discuss in more detail how we can conceptualize R, H, and E in terms of probabilities, and how those probabilities connect to our data. The exposure below pertains to a single location (i.e. grid cell in the rasterized map). In Section 2.2 we operationalize the theory by linking it to the NK and TR studies.

We can define a normalized measure of exposure E as a probability: $$E=P(visit)$$. Following the same logic, we can define a normalized measure for H as the conditional probability of a tick bite given a visit: $$H=P(bite|visit)$$.Then, we can define *R* as the unconditional probability of getting a tick bite: $$R=P(bite)$$. From the law of total probability, and the obvious fact that a tick bit requires a visit, we have: $$P(bite)=P(bite|visit)P(visit)+P(bite|no\,visit)P(no\,visit)$$$$=\,P(bite|visit)P(visit)\,$$, from which we recover R = H × E.

### Operationalisation

Let *v* be a variable representing the unknown total number of visits to each grid cell, and let *n* be the total number of records in the NK and TR studies, then an estimate of $E=P(visit)$ is $v/n$. The probability $H=P(bite|visit)\,\,$can be estimated as the total number of reported tick bites *b* at the location, divided by the number of visits $b/v$. Note that *H* may be zero when *b* = 0, and is undefined when *v* = 0. The unconditional probability: $R=P(bite)$ can be estimated by dividing the total number of tick bites by the number of records in the NK and TR studies $b/n$.

Using these estimates for the probabilities, we find that *v* can be calculated when the number of person-days in the study (i.e. the number of tick bite reports), and a measure of the hazard of the active ticks are known:

1 $$E=\frac{R}{H}=\frac{b}{nH}\,$$

Hence, it is possible to obtain a measure of human exposure to ticks by dividing the number of tick bites in a location by a measure of the hazard for that location. For a proxy of H, we use a previously developed hazard model^12^. Note that the value of *n* is immaterial as it is a constant over the Netherlands, and the output of the hazard model is not *H* but assumed to be proportional to *H*. The above is conditional on *H* being not equal to zero, which is satisfied since a property of our hazard model is that it always yields strictly positive hazard.

### Data processing

The calculation of human exposure to ticks requires calculating risk and hazard. The risk of getting a tick bite can be estimated from the volunteered tick bite reports, and hazard can be derived from a tick activity model developed in our previous work^12^. The workflow designed to obtain human exposure to ticks consists of four steps: (1) mapping the risk of getting a tick bite; (2) estimating tick hazard; (3) calculating and mapping human exposure; (4) validating the results. Note that this work has been developed using different Python libraries: numpy^21^ has been used to handle the different arrays, jenks^22^ package implements Jenks Natural Breaks (JNB) algorithm to classify the exposure layer, GDAL^23^ and cartopy^24^ were used to process geospatial data and prepare the maps, and matplotlib^25^, and seaborn^26^ to prepare the plots.

**Figure 1:**
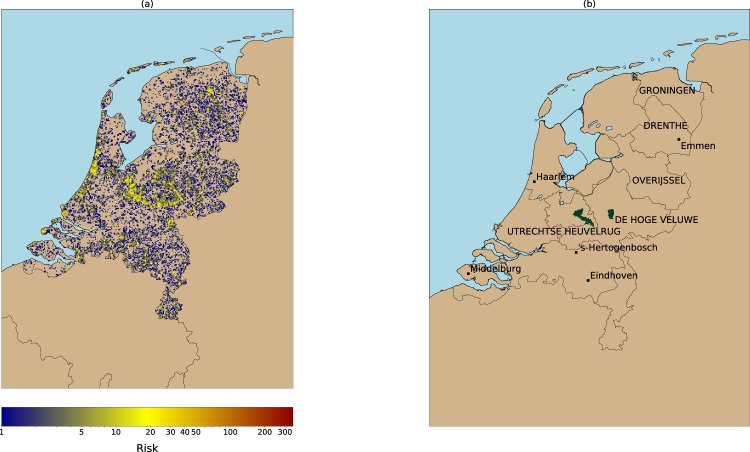
(**a**) Risk of tick bites (2006–2016) as collected by the NK and TR volunteered projects. The cumulative sum of tick bites reports per 1 km grid cell are used as a proxy of tick bite risk. The image reveals that tick bites are produced throughout the country. However, the reports tend to be clustered around forests (e.g. Veluwe national park, center of the country), or recreational areas (e.g. coastal areas). (**b**) Locations mentioned along the text. The provinces and the national parks are labeled with capital letters, whereas cities are labeled in lower case.

The transformation of tick bite reports into a risk layer requires selecting a spatial aggregation unit. Here we choose a regular grid with cells of 1 km^2^ as spatial unit because the existing hazard model works for grid cells with this spatial resolution. Thus, the tick bite reports were aggregated to grid cells of 1 km^2^. Risk is defined as the cumulative sum of tick bite reports over the period 2006–2016 occurring in each grid cell (Fig. 1a), if the cumulative sum is greater than zero.The number of tick bites per cell ranges between 1 (in blue) and 353 (in red). The visual inspection of Fig. 1a shows that coastal (e.g. from Haarlem to Middelburg), and forested areas (e.g. Veluwe national park, Utrechtse Heuvelrug) in the center of the country present a high concentration of high risk locations. These are well-known locations for outdoor recreation. Smaller regions of high tick bite risk can be found in the rest of the country (e.g. provinces of Drenthe and Groningen).

**Figure 2:**
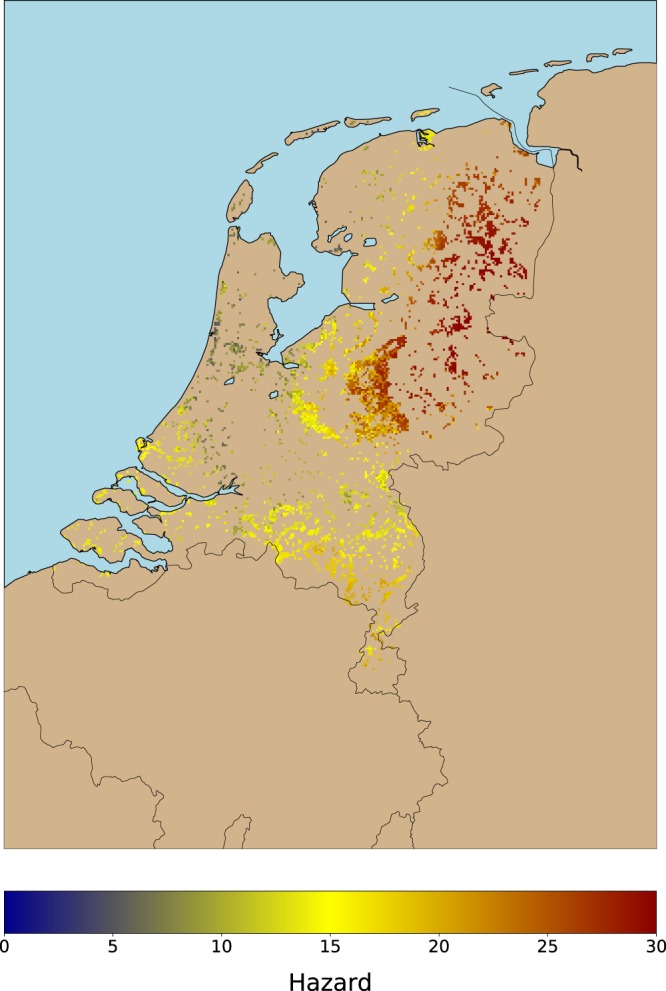
Hazard (e.g. tick activity) per 1 km grid cell. We used the model developed in^12^ to predict daily tick activity for the period 2006–2016. Then, we calculated the maximum mean tick activity for the period to devise this map. The numbers in the legend indicate the average number of active questing ticks per grid cell. The locations of the highest hazard are within the provinces of Groningen, Drenthe, and Overijssel, whereas the lowest hazard levels are located along the coastal areas.

Hazard is defined as the tick activity provided by a data-driven model^12^ that predicts daily tick activity in vegetated areas suitable for ticks (i.e. forests and natural grasslands). This model was built using nine years (2006–2014) of volunteered tick activity data (acquired in the Netherlands by using cloth dragging) and a large suite of environmental variables. Volunteers sampled 15 vegetated locations on a monthly basis, and counted the number of ticks per life stage (i.e. larvae, nymph, and adult). Our model, which was only calibrated for nymphs as they pose the highest hazard to humans, uses 101 biotic and abiotic environmental predictors. These predictors include data about the habitat conditions for ticks (e.g. litter, moss), mast years for three tree species, weather (e.g. temperature, evapotranspiration, relative humidity), satellite-derived vegetation indices (e.g. NDVI), and land cover. To account for the effect that short- and long-term weather conditions have on tick activity, we aggregated the weather data to 11 temporal resolutions (i.e. 1–7, 14, 30, 90, 365 days). The model yields daily tick activity for 4,132km^2^ for forests and grasslands (hereinafter ‘forests’), which enables further studies in the fields of ecological research, nature management and public health. Limitations of this model include the lack of data about wildlife, due to unavailability of this type of data at the national level and for the entire study period. Hazard predictions are included in this work by running our data-driven model for each day of each year included in the study period to compute the maximum annual tick activity, and by averaging these annual values to obtain a robust proxy of tick hazard at 1 km (Fig. 2). A visual inspection of Fig. 2 shows that the area of highest tick activity is located in the northeastern half of the country (e.g. provinces of Overijssel, and Drenthe). Forested areas in the center and south of the country present an average tick activity and coastal areas present a low tick activity.

As explained in section 2.2, human exposure can be calculated by dividing the risk and the hazard components. Note that there are locations in which these measures are not available. This means that there are locations in which no tick bites are reported, or locations outside forests with no measurement on tick activity available that are excluded from the exposure calculation. Depending on the values of these variables, four cases will be found (c.f. Table 1): (1) risk and hazard are positive (i.e. R > 0 and H > 0); (2) risk is positive and hazard is undefined (i.e. R > 0 and H = undefined); (3) risk is zero and hazard is positive (i.e. R = 0 and H > 0); (4) risk is zero and hazard is undefined (i.e. R = 0 and H = undefined). Cases 2 and 4 lead to a mathematically undefined operation, hence the exposure is undefined too. Case 1 represents locations in which there is tick activity and human exposure. Case 2 can be used to characterize locations where tick bites are reported outside forests (e.g. urban and peri-urban areas). Case 3 depicts forested locations with a low recreational intensity or a low hazard (i.e. no tick bites reported), and case 4 shows areas in which there is no risk and hazard data available.

**Table 1:** The four possible cases that can occur when dividing risk by hazard.

Case	Risk	Hazard	Exposure	Interpretation	Representation
1	R > 0	H > 0	E > 0	Standard case	Fig. 3, classified exposure (i.e. low, medium, high)
2	R > 0	H = undefined	E = undefined	Tick bites reported in non-forested locations	Fig. 6, light green
3	R = 0	H > 0	E = 0	Forest with a low intensity of recreation or hazard	Fig. 6, yellow
4	R = 0	H = undefined	E = undefined	No data	Fig. 6, grey

To ease the interpretation of the results, the resulting exposure was classified using the JNB algorithm. This algorithm minimizes the intra cluster variance and maximizes the distance between clusters. The optimal number of classes is iteratively found testing all values between 2 and 10 and calculating the goodness of variance fit of the resulting classification. Using the classes yielded by this algorithm, we created a categorical human exposure to ticks map.

To assess the validity of our exposure results, we compared them with a publicly available map depicting the attractiveness of the landscape (i.e. *Belevingswaarde van het landschap*, https://data.overheid.nl/data/dataset/dank-belevingswaarde-van-het-landschap, last accessed July 5^th^, 2018)^27,28^. The attractiveness map relies on 3 positive variables (i.e. naturalness, terrain elevation, historical value), and 3 negative variables (i.e. visibility of the horizon, urbanization, noise pollution) to classify the attractiveness of each grid cell. In short, this map shows how much citizens find a landscape attractive, expressed as six categories in the range 6–8. The less attractive locations have an attractiveness lower or equal to 7, and the most attractive ones an attractiveness higher than 7. For our validity assessment we first extracted the value of landscape attractiveness for each of the forested locations with calculated exposure. Then, we counted the number of grid cells in our exposure map that belong to each of the six attractiveness classes. Finally, we normalized these counts by the total number of grid cells belonging to each attractiveness class to obtain the percentages of tick bites that occurred in each exposure class.

## Results

**Figure 3:**
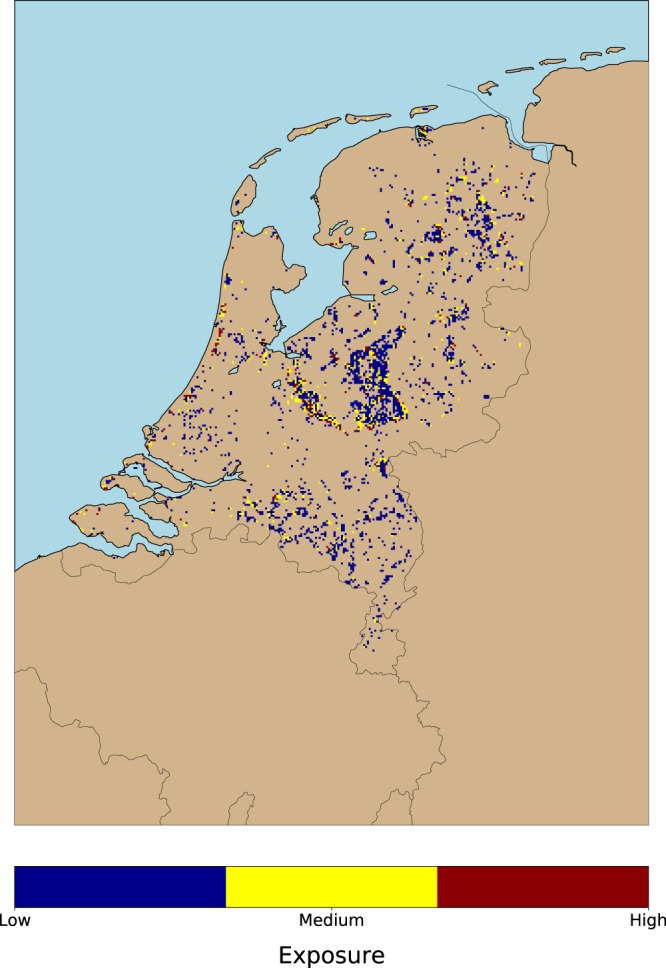
Human exposure to tick bites as a result of combining the maps in the previous two figures. Background color refers to non-forested locations or locations without tick bite reports. The exposure is classified in three categories. Well-known forest edges (e.g. Veluwe national park, Utrechtse Heuvelrug forest) and popular outdoor recreational areas (e.g. coastal areas from Haarlem to Middelburg) are classified as places with a medium and high human exposure. The remaining low exposure class depicts locations less intensely visited by people. Both results suggest that human exposure to tick bites is driven by two types of users (i.e. recreational, residential) as spotted in previous works^13,34^, that may require different treatment in the design of public health campaigns targeting a decrease on tick bites occurrence.

Figure 3 shows the classified tick exposure map, obtained by dividing the risk of getting a tick bite risk (Fig. 1a) by the hazard (Fig. 2). The application of the JNB algorithm resulted in the identification of three exposure classes: low, medium, and high. A visual inspection of Fig. 3 shows that there is a high amount of grid cells belonging to the medium exposure class. Those cells are especially concentrated along the forest edges of the Utrechtse Heuvelrug forest and of the Veluwe national park (center of the country). The class high exposure corresponds to highly popular places for outdoor activities, such as the coastal areas from Haarlem to Middelburg, or with a lower intensity, areas close to Hertogenbosch, Eindhoven, and around the small forest patches between Groningen and Emmen (north of the country). The class low exposure indicates locations that are less visited, and yet visitors could get bitten by ticks.

**Figure 4:**
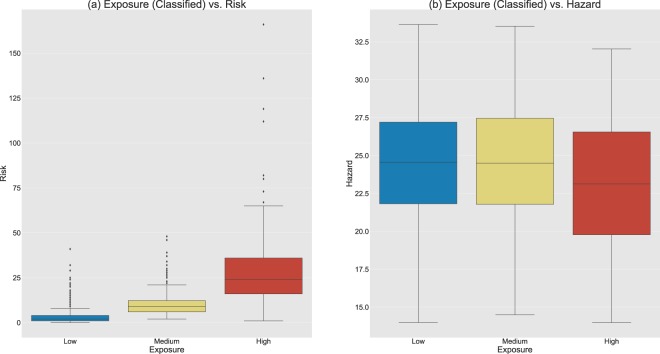
Boxplots showing the relationship between exposure and risk (**a**) and the relationship between exposure and hazard (**b**). For both figures, the X-axis shows the exposure class, and the Y-axis shows the number of tick bites per grid cells (**a**) and the tick activity per grid cell (**b**) respectively. Risk is a skewed distribution (i.e. long-tailed), thus presents low averages per boxplot, and a high number of outliers (reaching the maximum of 353 tick bite reports/cell), whereas hazard is a Gaussian-like distribution and so the averages per boxplot occupy the central part of the distribution. Plot A shows how the risk increases as the exposure increases, and plot B shows how the hazard remains (almost) constant as long as the exposure increases. This means that the risk of getting tick bites is mainly driven by exposure factors, regardless of the amount of hazard (e.g. tick activity) in a location.

Figure 4 explore the relationship between E, H, and R. The boxplot in Fig. 4a show that the risk of getting a tick bite has a skewed distribution (i.e. long-tailed distribution) spanning up to 353 tick bites per grid cell (not shown due to visual cluttering of the box plots), regardless of the exposure class. The medians of the boxplots (plot A) correspond to 3, 8, and 23 tick bites per km^2^ for the low, medium, and high exposure classes. The height of each box indicates the variety of risky conditions in which tick bites ensue. Low and medium exposure classes present a narrow range of risky conditions, whereas the high exposure class occurs in a wider range of conditions. The boxplots in Fig. 4b show that the hazards have a unimodal distribution, regardless on the exposure class. The medians indicate that tick bites occur in locations with similar levels of hazard, and the fairly uniform height of the boxes show that the range of risky conditions in which tick bites occur is alike. Note that Fig. 4a shows that the risk increases as the exposure increases, whereas Fig. 4b shows how hazard is almost constant as long as the exposure increases.

**Figure 5:**
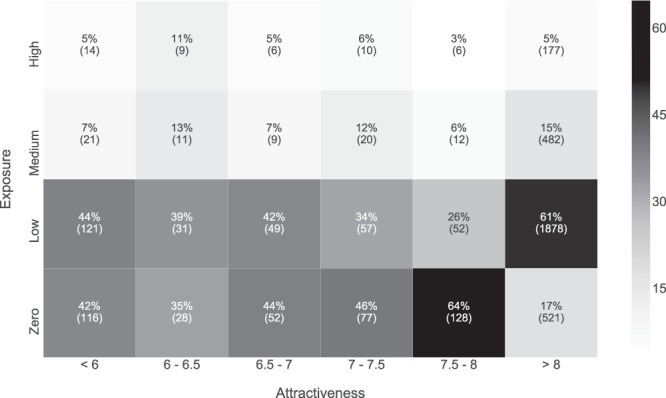
Heat map showing the relationship between the exposure classes and the attractiveness classes. The X-axis represents the six classes available in the attractiveness map, and the Y-axis the three classes of the exposure map. Thus, each cell in the heat map represents the number of grid cells belonging to both categories. Note that we applied a per-column normalization of the raw numbers to percentages to ease the interpretation of results, but both values are shown. The first three columns correspond to forest patches that are less attractive for citizens, whereas the last three columns correspond to attractive forested and rural locations. Thus, the first group of columns show a more urban exposure to ticks, whereas the second group of columns show human exposure to ticks in forested locations. The last two columns show an interesting pattern. The fifth column shows that 65% and 26% of the grid cells in the attractiveness class 7.5–8 have a zero and low exposure, respectively. The last column shows that 17% and 61% of the grid cells in the maximum attractiveness class have a zero and low exposure, respectively. This means that within forested locations, citizens have a preference for visiting a subset of them. Absolute numbers show that the majority of the exposure grid cells are concentrated along the column with the maximum attractiveness. This indicates that human recreational intensity is mainly concentrated in very appealing locations.

**Figure 6:**
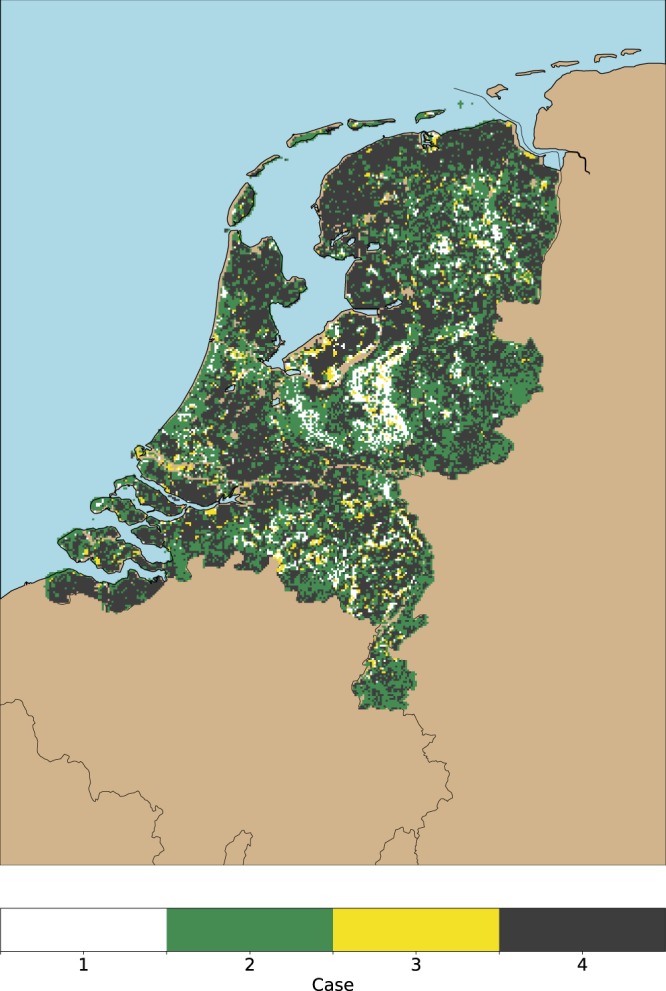
Visual representation of the four cases described in Table 1. To avoid visual cluttering, the classes in the first case (i.e. R > 0 and H > 0) have been condensed in one category (white). The remaining cases, namely, tick bites reported outside forests (i.e. R > 0 and H = undefined), forests with a low recreational intensity (i.e. R = 0 and H > 0), and locations with zero tick bites reported (i.e. low exposure or low hazard) R = 0 and H = undefined), are shown in the image in light green, yellow, and grey respectively.

Figure 5 shows the relationship between human exposure and the attractiveness of the landscape. Each cell represents a number of grid cells belonging to both categories. To ease the interpretation of results, note that we included the locations in which the exposure or the hazard are low (i.e. no tick bites registered in that forested location), and also that we applied a per-column normalization to turn the raw numbers to percentages. The visual inspection of the attractiveness layer and Fig. 6 reveals that the first three columns show the exposure in forest patches which are less attractive for citizens (i.e. ≤7), whereas the last three columns correspond to attractive forested and rural locations (i.e. >7). As seen, the distribution of exposure grid cells within the first group is similar among the different attractiveness classes. In the second group, there are two columns showing interesting patterns. The fifth column shows that 65% and 26% of the grid cells in the attractiveness class 7.5–8 have a zero and low exposure, respectively. The last column shows that 17% and 61% of the grid cells in the >8 class have a zero and low exposure, respectively. This indicates that citizens prefer visiting certain forested locations. The absolute numbers in Fig. 6 show that the majority of the human exposure is concentrated along the column with the maximum attractiveness. In Table 2 we provide a summary on the area in which humans are exposed to ticks. As seen, citizens are exposed to ticks in 271 km^2^ of forests in unattractive locations, and 2,694 km^2^ of forests in attractive locations. Note that there are 922 km^2^ of forest in which the exposure is zero. Thus, this study shows that citizens are more exposed to ticks in locations that are very appealing to the general public.

**Table 2:** Forested areas per human exposure and simplified landscape attractiveness classes (i.e. low corresponds to scores ≤7, whereas high to scores >7).

Exposure	Landscape attractiveness	
	Low	High
High	29 (6%)	193 (6%)
Medium	41 (9%)	514 (15%)
Low	201 (43%)	1,987 (58%)
Zero	196 (42%)	726 (21%)

Areas as expressed in km^2^ and as percentage over the total forested areas. There are 2,965 km^2^ of forests in which citizens are exposed to ticks, and 992 km^2^ with an exposure equal to zero (i.e. no tick bites recorded, although there might be a certain tick activity in those areas).

Figure 6 shows the four possible cases (Table 1) that can occur when dividing the risk by the hazard layer. To avoid visual cluttering, the three exposure classes from Fig. 3 have been merged into a single class. Figure 6 also shows the locations with tick bites outside forests, forests with a low recreational intensity, and the locations with zero tick bites reported. The visualization of cases 2 and 3 (Table 1) reveals two new insights. First, we can see that the occurrence of tick bites is a pervasive phenomenon that goes beyond the forest edges, because there are tick bites reported out of this scope, and across the country. Thus, vegetated landscape types (e.g. residential areas close to forest, urban parks with a dense tree coverage, natural coastal dunes with dense shrubs), might be optimal locations in which ticks and humans are in close contact. Second, there is a number of small forest patches without tick bite reports, which indicates that citizens do not intensively visit these locations.

## Discussion

Our results show that clusters of high human exposure are concentrated along forest edges and popular places for recreation. The analysis reveals that the exposure categories in Fig. 3 mostly occur in forested locations that are very attractive locations, as seen in Fig. 5 and Table 2. This can be related to previous literature, since transitional vegetation (i.e. ecotones) has been identified as a risky place to acquire tick-borne pathogens: humans tend to carry out outdoor activities along the forest edge, rather than going inside^29,30^. Moreover, forest edges are suitable locations for mammalian species to forage, and present higher abundances of ticks^31,32^. The Netherlands is heavily urbanized^33^, which means that multiple land uses are intertwined in a small area unit, thus bringing humans and ticks in peri-urban and residential areas in close contact. All the above suggests that the exposure to tick bites is driven by two types of activities, namely recreational, and residential, as suggested in previous works^13,34^. Therefore, we suggest defining different LB prevention campaigns and public health policies for each activity. For an instance, activities such as gardening^16^ can be linked to the residential exposure, whereas other activities such as scouting^13^, or outdoor sport^15^ competitions could be linked to the recreational exposure. Note that a limitation of this work is that we are unable to provide a measure of occupational risk, but farmers^35^, veterinarians^36^, landscapers^37^, or forest workers^38^ are known to have an elevated risk of LB infection. Unfortunately, information on whether the tick bite was acquired during a work-related activity was only available for the TR data and is therefore not incorporated in the model. However, these collectives tend to present a higher seroprevalence for LB^35,36^.

The boxplots in Fig. 4 show that the risk of getting a tick bite increases as the exposure increases, whereas the hazard remains constant as long as the exposure increases, which is indicative that the risk of getting a tick bite is driven by the human exposure in a location more than to the existing hazard. This makes sense, because humans might be unaware of the hazard, and do not consider this threat when organizing outdoor activities. For example, the recreational coastal areas between Haarlem and Middelburg present a relatively low hazard (Fig. 2), however, the risk of getting a tick bite (Fig. 1) is very high, because the human exposure around the area is high as well. This is also supported by Fig. 5 and Table 2, as they show that citizens mainly get exposed to ticks in areas which are very attractive, and therefore, it is likely that they have high numbers of recreational visitors.

These new insights show that hazard maps alone are insufficient to identify locations with a high risk for LB, motivating the creation of human exposure maps for public health specialists and forest managers. In this line, maps like the one presented in Figs 3 and 6 may help to facilitate the cooperation between public health specialists and foresters to implement prevention campaigns. For instance, these maps can be used to classify patches of forests that require active management and those that can suffice with only public awareness campaigns. In this work we encountered three main hurdles. First, the difficulty of validating the exposure results, since exposure heavily depends on the quality of the hazard model, and on the representativeness of the tick bite reports. The hazard model can capture general tick dynamics. However, hazard predictions might be uncertain in locations in which atmospheric conditions are not the main driver of tick activity. At the same time, the risk map contains an unknown factor of citizen’s reporting errors (e.g. positional inaccuracy at the time of adding the tick bite report to the NK and TR platforms, or citizens that over- or under-report tick bites). To overcome these issues and to validate the exposure products ICT data (e.g. mobile phones locations), geolocated data streams from social networks (e.g. Twitter) could be incorporated in the analysis. However, the use of this data is limited by privacy laws. Second, the exposure remains unknown in locations where there is no data from hazard or risk. This means that locations in which the hazard model is unable to predict the tick activity (e.g. non-forested areas), or locations where there are no tick bites registered, it is not possible to apply eq. 1, thus we cannot estimate human exposure to ticks. Third, there is a substantial number of locations in which there are no tick bites reported during the study period. We do not think that the risk is actually inexistent in these locations, but with the current data collections we are unable to disentangle if the zero tick bite count is due to a low human exposure or a low hazard. As a consequence, we choose to exclude these locations from the exposure calculation, because it is hard to assess whether or not it is a true zero, and subsequently we do not know if it is a robust indicator for low risk of tick bite.

## Conclusion

In this paper we present a first-of-its-kind map of human exposure to ticks in forested areas created from volunteered data. This map will hopefully contribute to mitigate the number of tick bites and hence of LB cases because exposure information might encourage forests managers and public health specialists to implement preventive measures. For instance, this map could be used to design targeted informative campaigns in recreational locations. Moreover, ecologists might use exposure information to locate hot spots of human disturbance, which could support better nature management practices. Future work should aim at quantifying uncertainties in the risk, hazard and exposure information, studying the main drivers of tick bites at each location (i.e. high hazard, vs. high exposure areas), and analyzing exposure in residential areas.

## Data Availability

The exposure data produced in this study are available from the corresponding author upon request.
